# Heritability of Directional Asymmetry in *Drosophila melanogaster*

**DOI:** 10.4061/2009/759159

**Published:** 2009-09-13

**Authors:** Ashley J. R. Carter, Elizabeth Osborne, David Houle

**Affiliations:** ^1^Department of Biological Science, Florida State University, Tallahassee, FL 32306-1100, USA; ^2^Department of Biological Sciences, California State University Long Beach, CA 90840, USA; ^3^Department of Psychology, Arizona State University, Tempe, AZ 85287-1104, USA

## Abstract

Directional asymmetry (DA), the consistent difference between a pair of morphological structures in which the same side is always larger than the other, presents an evolutionary mystery. Although many paired traits show DA, genetic variation for DA has not been unambiguously demonstrated. Artificial selection is a powerful technique for uncovering selectable genetic variation; we review and critique the limited number of previous studies that have been performed to select on DA and present the results of a novel artificial selection experiment on the DA of posterior crossvein location in *Drosophila* wings. Fifteen generations of selection in two genetically distinct lines were performed and none of the lines showed a significant response to selection. Our results therefore support and reconfirm previous findings; despite apparent natural variation and evolution of DA in nature, DA remains a paradoxical trait that does not respond to artificial selection.

## 1. Introduction

Directional asymmetry (DA) is the consistent difference between a pair of morphological structures, for example, the larger of the pair occurs consistently onone side. Symmetry in external morphology is the rule among metazoans, making the cases of DA particularly striking. Good examples of such asymmetries include the enlargement of the left tooth of the narwhal to form a “horn,” the morphology of flounders, and the absence of the left lung in some *Gymnophiona* species. Many instances of more subtle quantitative instances of DA have also been observed, such as testicle position and size in mammals and wing size and shape in Diptera. In contrast, internal organs, particularly those that are not paired, such as the vertebrate heart, very often show asymmetry.

Evidence for additive genetic variation for DA has been unusually difficult to demonstrate, leading many authors to treat DA as the canonical example of a trait without heritable variation (e.g., Lewontin [1, page 92]). Several experiments (reviewed later) have attempted to select for DA in different traits in *Drosophila*, but only one [2] showed a small significant response. In contrast, virtually all other morphological traits readily respond to artificial selection [1, 3]. The apparent lack of selectable additive genetic variation for a trait that has clearly evolved in nature is an evolutionary mystery that deserves more attention. 

Demonstrating absence of genetic variation is of course impossible by statistical means, although we can set upper bounds on its magnitude. As detailed below, previous studies of response to selection on DA are not numerous, and many studies did not allow the estimation of such limits. 

Three hypotheses have been proposed to explain the relative inability of DA to evolve under artificial selection. The standard explanation for DA's lack of evolvability is that no left-right axis exists, but the prevalence of asymmetry in internal morphology as well as the very widespread existence of subtle DA in insect wings [4] clearly reveals that this general proposition is untenable. A more sophisticated version of this “absent axis” hypothesis is that all bilaterally symmetric organisms possess an axis that specifies distance from the midline that generates that symmetry. DA then requires an additional mechanism to break that symmetry, and that mechanism is difficult to evolve, perhaps because the number of genomic changes that can break symmetry is very small. Finally, Palmer [5] has suggested that DA is more likely to evolve from a state of antisymmetry, where modal individuals are asymmetrical but the direction of asymmetry is random, than from a state of symmetry. Under this hypothesis, DA is difficult to evolve without first decanalizing the symmetry found in most organisms. 

Given this debate, Dipteran wing morphology is an intriguing target for selection on DA. Large qualitative DA exists in males of *Erebomyia exalloptera* [6]. Pélabon and Hansen [4] recently reviewed over 100 papers that test for DA in wing size in insects, and over one-quarter of the statistical tests and one-third of the species show significant DA, although the average magnitude is less than 1% of overall size. Klingenberg et al. [7] found consistent but very small DA in representatives of three different Dipteran families, including *Drosophila melanogaster*, an observation that has since been confirmed in two other *Drosophila* species [8, 9]. These results suggest that a left-right axis has previously evolved and therefore genetic variation for left-right positioning is more likely to exist in wing traits than in others. A search of the scientific literature for reports of directional asymmetry selection experiments revealed only five such studies, all using *Drosophila*.

The earliest published DA selection experiment was conducted by Maynard Smith and Sondhi [10]. They selected on the ocelli and associated bristles in *Drosophila subobscura*. Individual flies can possess up to three ocelli, one central and anterior and two posterior ocelli. In their “symmetrical” SYM line, flies with both the left and right posterior ocelli were selected, and in the “asymmetric” ASY line, flies with both anterior and left posterior ocelli, but without the right ocellus, were selected. This selection treatment thus favored individuals with a leftward bias in ocellus placement. Both lines were selected for 12 generations, with over 200 flies measured and 20–30 selected for mating each generation. In the SYM line, they observed more than a fourfold increase in the frequency of the selected symmetric phenotype (14.75% to 64.20%), but the change was almost entirely explainable by loss of the anterior ocellus. In the ASY line, they observed a threefold increase in the frequency of the selected asymmetric phenotype, but this was almost entirely explainable by an increase in anterior ocellus prevalence. The frequency of flies possessing only the left posterior ocellus fluctuated around a frequency of 0.5 with no trend. Other, single-generation crosses showed no apparent inheritance of the handedness of ocellus presence. Maynard Smith and Sondhi concluded: “It seems likely that the handedness of individuals is a purely chance phenomenon in the sense that it is not influenced either genetically or maternally.” [10].

Beardmore [2] selected on DA of sternopleural bristle number in *Drosophila melanogaster*. The selection index was the ratio of the number of bristles on the two sides, left over right, which was selected for increase in one line and decrease in the other. To avoid selection for increased mean bristle number, if two flies possessed the same ratio, the investigators selected the fly with the lower total bristle number. All lines were selected for 50 generations; 60 flies were measured, and 30 selected for mating in each generation. Beardmore also maintained one control line that was randomly selected. Regression of divergence between the two lines on generation showed significant divergence in directional asymmetry at *P* = .03. He estimated a heritability of 3.6%, but this involved an arbitrary assumption that the selection differential was half of that imposed. Additionally, values from his control population resembled those from the decrease line, casting some doubt on the reliability of these conclusions. Furthermore, changes in overall bristle number were not reported, despite his selection for decreased overall bristle number. Selection for smaller total bristle number may have generated larger bias values if the mean number decreased. Although Beardmore concluded that heritable variation for DA was present, his quantitative estimates are questionable.

**Figure 1:**
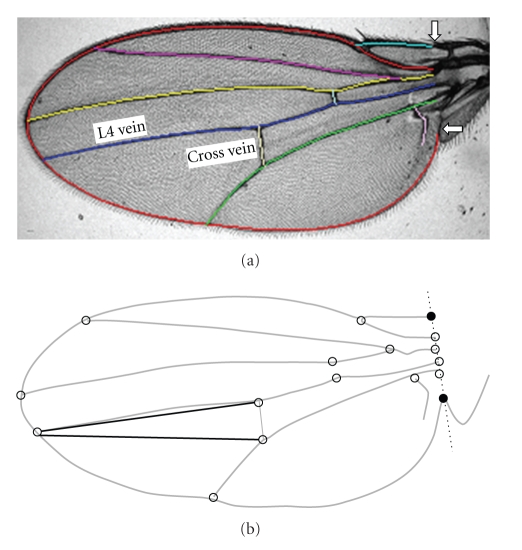
Image of splined *Drosophila melanogaster* wing and distances used in study. (a) Original photographic image of wing with B-spline overlay. White arrows indicate user-defined landmarks used by the B-spline algorithm and two veins used in distance calculations labeled. (b) Wing-vein intersections shown with the two distances used in this study indicated by heavy black lines.

Purnell and Thompson [11] selected for DA of wing folding in *Drosophila melanogaster*. At rest, a *Drosophila*  individual holds its wings against its back with one over the other, always crossing the same wing over the other throughout its life. Purnell and Thompson created two selection lines, right-over-left folding and left-over-right folding. Each line was selected for 15 generations; 50 flies of each gender were assayed, and 5 selected for mating in each generation. Their analysis of the entire experiment shows a nonsignificant response in both lines, with an average heritability of −5.8%. Purnell and Thompson claimed to have found a significant response in the early generations of their experiments, but examination of their [11, Figure  1] suggests that this conclusion was based on an ad hoc choice of time periods. 

Coyne [12] selected for DA of eye size for 30 generations in a population carrying the mutant eyeless allele (ey^r^) backcrossed into an outbred population of *Drosophila melanogaster*. Eyeless reduces facet number and simultaneously decanalizes symmetry, creating variation in facet number asymmetry that Coyne could judge qualitatively. As Coyne noted, he was more readily able to judge asymmetry in flies with one very small (or missing) eye. Three selection lines were created: left eye larger, right eye larger, and both eyes smaller—plus an unselected control. Each line was selected for 30 generations; 200–500 flies were assayed and roughly 10–20% selected for mating in each generation. At the end of the experiment, Coyne tested whether the ratio of left-eyed to right-eyed flies differed from 0.5. There was no evidence for a response in directional asymmetry, and the one significant deviation from 0.5 was in a control line. Unfortunately, Coyne's use of qualitative data precluded estimation of an upper limit to heritability [12]. Also unclear is whether the DA present in a decanalized mutant stock is relevant to variation in natural populations. 

Tuinstra et al. [13] selected on DA in an outbred *Drosophila melanogaster* population fixed for a *scute* mutation that decanalizes scutellar bristle number. They performed a combination of family and individual selection for the difference between left and right bristle numbers. Each line was selected for 12 generations; 16–18 sibships were assayed, 4–6 sibships provided the males, and 4–6 other sibships provided the females for mating in each generation. No significant response was found in various measures of directional asymmetry. The realized heritability (±S.E.) for their selected index was negative in both sexes (−.006 ± .105 in females, −.055 ± .175 in males) and not even close to statistical significance. The experiment was terminated after 12 generations of selection because of severe infertility. This problem suggests that either the initial population was inbred or it became inbred because of the small population size; either of which would reduce the likelihood of detecting genetic variation in DA.

Despite the results of the experiments described above, there is other evidence suggestive of the presence of genetic variation in insect wing DA. Pélabon et al. [14] selected for different shape of left wings in *D. melanogaster* for nine generations, obtaining large direct responses in shape. They found that DA was present in the controls and also underwent consistent changes in magnitude under selection, even though populations were selected in opposite directions. Rego et al. [15] found substantial increases in DA in wing size and shape in hybrids between *D. subobscura* and *D. maderiensis*, and that the degree of DA differed among families within the hybrid population. Santos et al. [9] found that DA in wing shape and size differed significantly in some cases between karyotypes and crosses in *D. subobscura*, although none of these differences remains significant if corrected for multiple testing. All of these results indicate the existence of genetic variation that affects DA, but whether this generates additive genetic variance for DA within populations is not clear.

In order to investigate whether selectable genetic variation for DA may exist in insect wings we performed an artificial selection experiment on the DA in position of the posterior crossvein in the wing of two genetically distinct lines of *Drosophila melanogaster*. The position of this crossvein is easily quantifiable and has been shown to be variable within the species (e.g., by [14, 16, 17]) making it an ideal trait for this study.

## 2. Methods

### 2.1. Selection Lines

We performed artificial selection in two wild-type populations recently derived from the wild; iso-female lines founded with flies collected from Tallahassee, Florida (FL) and central Illinois (IL) were generated and maintained for less than six months prior to use in this experiment. We constructed the two populations used in this study by pooling 30 of these isofemale lines. One generation of random mating with a population size of approximately 300 was performed prior to the initial selected generation for each population. Prior to and throughout the course of the experiment, flies were cultured in 45 mL shell vials at 25°C and under a 12 : 12 light cycle on unyeasted standard corn-meal, sucrose, brewer's yeast medium. During the experiment, vials were initiated with five adult males and five adult females. Adults of each group were allowed to lay eggs for 48 hours in each of three vials and then discarded. Their offspring were collected as virgins using CO_2_ anesthesia 9–11 days later.

### 2.2. Wing Measurement

Wing measurements were made with the WINGMACHINE system [16]. For measurement, flies were placed under CO_2_ anesthesia; then each wing was briefly drawn into a suction device and a digital wing image obtained. The user digitized two landmarks on each wing, then the WINGMACHINE software automatically fit a B-spline model to the locations of all the wing veins. The parameters of this model were then used to calculate the appropriate selection index. Before selection, the images of wings with the most extreme DA values were rechecked for splining errors and resplined if necessary to ensure that gross measurement errors were avoided.

### 2.3. Selection Procedure

We used the relative position of the posterior crossvein on the left and right wings as our measure of DA. The location of the crossvein was measured as the average distance from the two ends of the crossvein to the end of vein L4 (see Figure 1). Distances on the left (*X*_L_) and right wings (*X*_R_) were used to calculate directional asymmetry via DA = *X*_L_ − *X*_R_. In each population, we selected one line to have positive DA (replicates L-FL and L-IL) and one to have negative DA (replicates R-FL and R-IL). Selection on the L lines sought to move the crossveins to the left (as viewed from above and behind when the wings are extended as in flight) and that on the R lines to move the crossveins to the right. Selection continued for 15 generations. Fifty flies of each gender were measured, and 15 were selected in each generation. Selected flies were randomly mated. The use of 30 isofemale lines preserves essentially all of the original populations' heterozygosities and much of the allelic diversities; although the design of this experiment did not include replication within lines to guarantee our selection as the cause of any single observed phenotypic response, the lack of a significant response in any of the treatments provides strong evidence against the presence of selectable genetic variation in the original populations.

Selection using identical conditions (population sizes and fraction mated) generated large phenotypic responses for wing vein traits in a separate set of experiments (more than 10 SE change in mean phenotypic index, data unpublished) with continued response for over 20 generations, loss of initial genetic diversity due to inbreeding is therefore unlikely to be a factor in this shorter experiment.

### 2.4. Data Analysis

During the analyses of variance reported below, three outlier individuals with DA residuals more than 5 S.D. from the mean were identified. Re-examination of the wing images and spline data showed that these did not result from splining errors or damaged wings and were genuinely unusually asymmetrical pairs of wings. These observations were omitted from analyses of temporal trends and DA distribution but included in analyses of realized heritability. For each of the 15 generations, the means of the selected individuals and overall population were used to calculate a selection differential (mean of selected—overall mean), *S*, and a selection response (mean of next generation—mean of current generation), *R*. Separate *S* and *R* values were calculated for males and females, and these were averaged to yield the input for the realized heritability analyses.

The realized heritability of DA was calculated using several approaches. In the first two, we assumed that the response in each generation was an independent variate. First, we estimated realized heritability as the slope of the linear regression of the response on selection differential for the single-generation estimates, without fitting an intercept. Second, we estimated realized heritability as the slope of the linear regression of the cumulative response versus cumulative selection differential. Although calculating realized heritabilities using these methods is accepted practice, the responses are not in fact independent, biasing statistical testing and confidence-interval estimation.

Our other approaches took explicit account of the sampling dependencies among generations by using a generalized least squares (GLSs) approach (see, e.g., Lynch and Walsh [18, page 202]). This approach incorporates a matrix of variances and covariances among the response variables. The GLS parameter vector, *b*, is estimated as

(1) $$b\;=\;{\left(X^{\text{T}}W^{-1}X\right)}^{-1}X^{\text{T}}W^{-1}R,$$

where *X* is the design or incidence matrix of predictor values (in this case a vector of selection differentials, with or without an intercept vector), *W* is a square matrix that incorporates the sampling variance of the selection responses on the diagonal and the sampling covariances in the off diagonals, and *R* is the vector of observed responses to selection. The standard error of *b* is

(2) $${\text{SE}}_{b}\;=\;{\left({\left(X^{\text{T}}W^{-1}X\right)}^{-1}\right)}^{1/2}.$$

GLS analyses were performed both on the cumulative selection differentials and responses and on the generation-by-generation differentials and responses. In the generation-by-generation case, the covariances in the *W* matrix between successive *R* values are negative and equal to the sampling error of the shared generation. All other covariances are 0. In the cumulative case, the covariances in the *W* matrix are all positive and equal to the sampling variance in the first generation, because all estimates of *R* are based on data from the initial generation. In the cumulative cases, we estimated an intercept, but the small values estimated are of little relevance to the interpretation of heritability, so we do not report them. Statistical analyses were performed in SAS 9.1 [19].

## 3. Results

**Figure 2:**
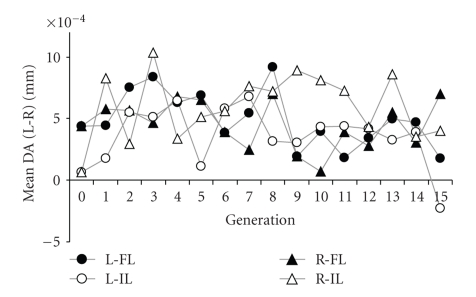
Mean directional asymmetry (DA) of each generation for four lines, two originally from Florida (FL) and two from Illinois (IL). The *y*-axis shows the mean DA (left-right) value in millimeters for the 100 individuals measured in each line in each generation. L indicates means for lines selected to move the crossvein left (to decrease DA value) and R those selected to move the crossvein right (to increase DA value).

Mean values of DA of posterior crossvein position for each generation are presented in Figure 2. Little response to selection on DA values is apparent over the entire experiment. A potential exception is the last generation, when the two leftward treatments were below the two rightward treatments, the only generation where this was the case. To test for temporal trends, we performed analyses of covariance on DA with population and selection treatment as fixed effects and generation as a covariate. Separate analyses were performed for each gender. The only significant effects without Bonferroni corrections were a treatment-by-population effect in females (*P* = .02) and a generation-by-population effect in males (*P* = .04). Inspection of the parameter estimates shows that the slope in the FL population was negative and that in the IL population was positive but that in neither population did the estimates differ significantly from 0. The generation-by-treatment effect tests for changes consistent with a response to selection, but these terms were not significant (*P* = .55 in females, *P* = .23 in males).

Given the lack of temporal trends, we computed descriptive statistics on all generations pooled (Table 1). The total sample size for these statistics is 3200 individuals and 6400 wings for each gender. Overall mean DA reflects a consistent rightward bias of 0.00048 mm, 0.3% of the mean posterior crossvein distance. Mean DA was 70% higher in females, whereas the mean crossvein position was only 15% larger. The variation in DA is similarly small; its S.D. is only 1.2% of mean posterior crossvein distance.

To examine the distribution of DA within generations, we pooled the residuals from each generation and line mean, omitting three outlier individuals. The overall distribution was well approximated by a normal distribution. Female residuals did not depart significantly from normal by the Cramer-von Mises test (*W* ^2^ = 0.101, *P* = .11), but those of males did (*W* ^2^ = 0.211, *P* < .005). The distribution was modestly leptokurtic, with remarkably similar positive kurtosis values in the two genders. These are highly significantly different from 0 according to tabled values. Positive kurtosis is consistent with the existence of true variation among individuals in their asymmetries.

To test for significant DA in the entire data set, we performed separate mixed-model ANOVAs on the crossvein position for the two genders (Table 2). Given the lack of trends in DA with time, we chose to treat generation as a categorical randomeffect. Analyses with population (IL or FL) as random effects and treatment (left or right) as main effects showed no evidence of main effects but complex interactions suggesting variation due to time of rearing and measurement. Because the IL and FL populations were raised and measured in alternate weeks, we cannot disentangle these effects in our data. In the analysis shown in Table 2, we treated generation and line (the combination of selection and population: L-FL, R-FL, L-IL, R-IL) as random effects, and side as a fixed effect. The “side” effect tests for DA and is significant at *P* < .0015 in both sexes. We therefore confirm the finding of a small DA in wing shape in *D. melanogaster*. We interpret the highly significant line-by-generation effect as a signature of random temporal variation in DA that could reflect rearing conditions or differences in the handling and measurement of flies.

**Figure 3:**
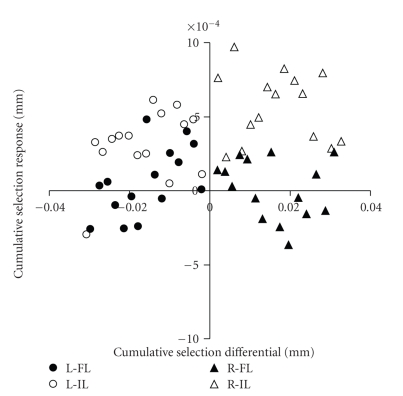
Cumulative selection differential versus cumulative response. Abbreviations as in Figure 2. Note that the position of the open symbols above the zero cumulative-response line is mainly due to the low first-generation value of the Illinois population (lines L-IL and R-IL).

To put the potential selection responses in a quantitative genetic framework, we estimated the realized heritability of DA in each line. The relationship between cumulative selection differentials and cumulative response is shown in Figure 3. Table 3 presents the realized heritability estimates for the ordinary least squares (OLSs) and generalized least squares (GLSs) linear regressions. Comparisons of the realized heritability estimates to their standard errors indicate that no significant response was observed in any of the lines (Table 3), with only line L-FL coming close. On the assumption that each population and treatment estimates the same parameter, the generation-by-generation GLS analyses yielded an overall estimate of realized heritability of .003 ± .003 and the cumulative GLS analyses .003 ± .005. Taking the less precise estimate, we can be 95% certain that the realized heritability of DA of crossvein position is less than 1.4%.

## 4. Discussion

We were unable to detect a response to selection in directional asymmetry (DA) in a wing trait in *Drosophila melanogaster. * Our best estimates of realized heritability were not significantly different from 0 and the confidence intervals indicate that the heritability of DA, if present at all, is very likely to be less than 1.4%. Directional asymmetry (DA) is often taken as an example of a type of trait, indeed perhaps the only phenotypically variable morphological trait, that lacks additive genetic variation. Our review of previous attempts to select for DA suggests that this conclusion rests on a somewhat shaky empirical foundation; our results firm up this foundation. 

Several hypotheses address the inability of DA to evolve readily under directional selection. One, discussed above, is the “absent axis” hypothesis, that is, there are no genetic variants capable of producing DA. A second hypothesis is that, even if such an axis does exist, the number of base pairs that affect it is very small. If the mutational target is small, the chances of finding a population with the genetic variation that would allow a response would also be small. We call this the “small target” hypothesis. Finally, Palmer et al. [5] have suggested that DA originating late in development is more likely to evolve from a state of antisymmetry, where modal individuals are asymmetrical but the direction of asymmetry is random, than from a state of symmetry. If so, the ability to evolve DA might depend on the preexistence of antisymmetry. If it does, then attempts to select directly for DA of adult structures in a symmetric ancestor might be ineffective because the proper genomic conditions have not been set up to allow expression of variation in DA.

The last hypothesis is supported by a comparative analysis of conspicuous asymmetries in paired structures [20]. The inferred ancestral states of symmetry in clades containing conspicuous cases of DA are almost as likely to be antisymmetry as symmetry, suggesting that DA often evolves from an antisymmetric state rather than a symmetric one. Given the global rarity of antisymmetry, this analysis would overestimate the proportion of time that the ancestor was symmetric. Antisymmetry may therefore be a necessary precursor to the evolution of DA. We call this the “antisymmetry first” hypothesis.

Our selection experiment is the first to be performed on a trait that already shows directional asymmetry and thus provides unique insight into the three hypotheses. As demonstrated by several previous authors [7, 8, 14] and confirmed by us, subtle DA appears to be generally present in the wings of Diptera. 

The existence of DA in the base population for our experiment clearly rules out the absent-axis hypothesis as an explanation for the failure of DA to evolve. The small-target hypothesis, in contrast, is strengthened by our results. Considerable progress has been made toward describing the genetic basis of asymmetries in internal organs, particularly in vertebrates. Described mutations affecting symmetry have qualitative effects on symmetry, in some cases generating a symmetrical morphology from a normally directionally asymmetric state, in others reversing the normal asymmetry, while in still others yielding antisymmetry [21]. Recently, a gene encoding one form of myosin has been shown to be capable of reversing asymmetries in the gut and genitalia of *Drosophila melanogaster* [22, 23]. The genetics of asymmetries of paired structures, and in particular those of external morphology, are much less well known. In the majority of cases where inheritance of bias has been looked for, it has not been found, although some evidence supports of inheritance of handedness in humans and eye side in flounder. This result is consistent with a small genetic target size.

A strict interpretation of the antisymmetry-first hypothesis cannot be supported by selection in a population that already possesses DA, but a *Drosophila* population with wing antisymmetry might still be more responsive to selection for DA than our populations. In a separate study (Carter and Houle, submitted), we have successfully selected for increased fluctuating asymmetry in wing morphology in *D. melanogaster *in a 43-generation selection experiment. Fluctuating asymmetries (FAs) are random deviations from perfect symmetry. As Palmer and Strobeck [24] emphasized, selection for FA is essentially selection on a variance. Selection for FA would therefore favor the expression of antisymmetry as well as increases in variance of other kinds. In the study by Carter and Houle (submitted), the heritability of FA was less than 1% but this is not directly comparable to that of DA, DA is a difference in mean position, while FA measures the variance in position. The heritability of FA is much less than the heritability of the underlying cause of genetic variation in variance. Nevertheless our ability to obtain a response to selection on FA, but not to selection on DA, is at least consistent with the idea that it may be easier for variance to evolve than for directional asymmetry due to a difference in mutational target size.

Overall, our results favor the small-target hypothesis, but whether this finding can be generalized to other traits or populations is difficult to know. Although DA has not been demonstrated for other traits subjected to artificial selection for DA, neither have those traits been tested for subtle DA. DA in wings is only apparent with very large sample sizes (as in our study) or high-quality multivariate data. Few traits have been subjected to this level of scrutiny.

DA is a condition that has clearly evolved multiple times, but its apparent lack of additive genetic variation is an evolutionary mystery that deserves more attention. The evolution of DA remains paradoxical.

## Figures and Tables

**Table 1:** Basic statistics of crossvein position and directional asymmetry (DA, ±S.E.). Crossvein position is the mean of the distances from the endpoints of the posterior crossvein to the end of vein L4 in the *Drosophila melanogaster* wing, ±S.E. Residual S.D. is pooled departures from the line and generation means.

	Male	Female
Crossvein position	0.140 ± 0.00009	0.161 ± 0.00011
DA	0.00036 ± 0.00003	0.00060 ± 0.00003
DA 1% quantile	−0.00364	−0.00396
DA median	0.00037	0.00058
DA 99% quantile	0.00471	0.00523
Residual S.D.	0.00169	0.00193
Residual kurtosis	0.589	0.575

**Table 2:** Mixed-model analyses of variance of crossvein position in males and females. Line and generation are treated as categorical random variables, and side (left or right) as a fixed effect. All tests of higher interactions had *P* > .5 and were omitted from these analyses. S.E. are standard errors from the covtest option in SAS Proc Mixed.

		Num.	Den.		Variance	S.E.	
Gender	Source	df	df	F	×10^6^	×10^6^	*P*
Males	Line				1.277	1.315	.166
	Generation				3.119	1.647	.029
	Line × Gen				5.104	1.119	<10^−4^
	Side	1	6331	10.1			.0015
	Error				20.18	0.358	
Females	Line				1.791	1.752	.153
	Generation				2.218	1.358	.051
	Line × Gen				3.557	1.189	<10^−4^
	Side	1	6335	19.9			<10^−4^
	Error				28.476	0.506	

**Table 3:** Realized heritability estimates (±S.E.) for DA obtained using four techniques. Data was either the single-generation values of selection differential and response or the cumulative ones. Heritabilities were calculated either with ordinary unweighted least squares (OLSs) regression or with a weighted generalized least squares (GLSs) regression.

	By generation		Cumulative	
Line	OLS	GLS	OLS	GLS
L-FL	0.013 ± 0.037	0.011 ± 0.007	0.015 ± 0.006	0.014 ± 0.008
R-FL	0.011 ± 0.033	−0.004 ± 0.007	−0.006 ± 0.006	−0.006 ± 0.007
L-IL	0.009 ± 0.037	0.004 ± 0.007	0.009 ± 0.008	0.010 ± 0.008
R-IL	0.010 ± 0.050	0.003 ± 0.007	−0.003 ± 0.006	−0.004 ± 0.008
